# Latent accelerated diffusion-based deformation estimation for real-time volumetric imaging

**DOI:** 10.1088/1361-6560/ae8127

**Published:** 2026-07-07

**Authors:** Kangning Zhang, Grace He, Yinheng Zhu, Qingying Wang, Mingli Chen, Weiguo Lu, Xuejun Gu

**Affiliations:** 1 Department of Radiation Oncology, Stanford University, Stanford, CA 94305, United States of America; 2 Department of Bioengineering, University of Illinois at Urbana-Champaign, Urbana, IL 61801, United States of America; 3 Department of Radiation Oncology, The University of Texas Southwestern Medical Center, Dallas, TX 75390, United States of America

**Keywords:** real-time deformation, real-time volumetric imaging, x-ray projections, lung SBRT

## Abstract

**Objective.:**

The proposed method aims to address the technical limitations in 3D respiratory estimation and image reconstruction from ultra-sparse views, overcoming data-acquisition constraints that often hinder conventional deformable image registration and volumetric imaging in real-time clinical applications.

**Approach.:**

We propose a Latent Accelerated Diffusion framework for Deformation Estimation enabling Real-time volumetric imaging (LADDER) framework, which integrates: (1) a deformation network (VoxelMorph) that generates a patient-specific baseline deformation vector field (DVF) from pre-treatment imaging (e.g. end-inhale to end-exhale), and (2) a latent diffusion model (LDM)-based deformable image registration (DIR) model that estimates DVF scaling factors and residual maps to generate intra-treatment real-time DVF and dynamic volumetric images. The LDM compresses the baseline DVF into a compact latent manifold, enabling fast, projection-conditioned refinement guided by anatomical cues. A physics-informed loss enforces anatomical regularity and consistency with measured projections. LADDER was trained on the Learn2Reg dataset and evaluated on 10 DIR-Lab lung datasets across compression factors, spatial down-sampling, projection configurations (single- vs dual-view), and baseline DVF spans.

**Main results.:**

With a dual-projection input, an end-inhale to end-exhale baseline DVF and a compression and down-sampling factor of 8, on 10 test cases, LADDER achieves a mean target registration error (TRE) of 0.87 ± 0.33 mm, and high volumetric structure similarity (3D SSIM > 0.95) and low volumetric reconstruction error (3D NMSE < 0.006), while maintaining real-time inference (~0.11–0.12 s). Further analysis shows the range of DVF span impacts deformation accuracy, and the dual-projection input improves deformation fidelity and reduced variability across breathing phases compared to the single-projection.

**Significance.:**

LADDER enables real-time 3D motion estimation and volumetric reconstruction from ultra-sparse x-ray views by conditioning diffusion in a patient-specific DVF latent space. Its submillimeter TRE and real-time speed indicate strong potential for next-generation motion management and image guidance, supporting on-the-fly anatomical modeling to enhance the safety and efficacy of lung stereotactic body radiation therapy.

## 1. Introduction

Stereotactic body radiation therapy (SBRT) for lung cancer represents a significant advance in radiation oncology, combining precise image guidance, motion management, and ablative high-dose delivery to eradicate tumors while minimizing exposure to adjacent healthy tissue and organs at risk (OARs) (Tsang 2016, Nagata and Kimura 2025). In early-stage, medically inoperable patients, SBRT consistently achieves excellent local control and favorable survival with a low toxicity profile and in selected operable cohorts, outcomes appear comparable to surgery (Timmerman *et al* 2003, McGarry *et al* 2005, Timmerman *et al* 2018). However, on treatment delivery, lung SBRT faces a major hurdle: respiratory motion can compromise dose precision, leading to tumor underdosing or healthy tissue toxicity (Keall *et al* 2006, Brandner *et al* 2017). This is especially critical in proton therapy, where anatomy deformation and target motion caused by respiratory motion can displace the Bragg peak off the target to the OARs. Current mitigation strategies (Keall *et al* 2006, Brandner *et al* 2017, Lansing *et al* 2026) include breath-hold (motion freezing), abdominal compression (motion impeding), ITV-based planning (motion inclusion), respiratory gating (motion gating). However, each approach carries inherent limitations, ranging from patient intolerance and prolonged treatment times to increased irradiated volumes and reliance on external surrogates susceptible to correlation errors. Ultimately, these strategies fall short of next-generation motion management, which requires both real-time volumetric imaging and on-the-fly deformation modeling to shift the focus from geometric tracking to precise dose management (e.g. dose tracking and dose adaptation).

Real-time volumetric imaging is close to clinical reality with the recent advancements in imaging detectors and computational power, but significant challenges remain (Pan *et al* 2025, Shao *et al* 2025). Traditional 4D-CBCT methods rely on retrospective binning hundreds of projections. This inherently limits total acquisition time to ⩾1 min, even with projection speeds below 100 milliseconds, making them too slow for real-time use and incompatible with irregular intra-treatment motion (Endo *et al* 2003, Sonke *et al* 2005). High temporal resolution (sub-second volumetric imaging) poses a fundamental trade-off between speed and sampling completeness. For x-ray-guided radiotherapy (RT), this necessitates reconstructing volumetric images from 1–2 real-time projections. Such ultra-sparse sampling yields a severely ill-posed spatiotemporal inverse problem that conventional back projection methods (Feldkamp *et al* 1984, Yu *et al* 2007), compressed sensing methods (Candes *et al* 2006, Chen *et al* 2008, Sidky and Pan 2008, Yu and Wang 2009, Choi *et al* 2010) and maximum *a posteriori* estimations (Fessler and Rogers 1996, Ji *et al* 2008) *cannot* reliably solve. Recent AI approaches, like NeRP (Shen *et al* 2019) and VolumeNeRF (Liu and Bai 2024) utilize implicit neural representations and patient-specific priors to reconstruct 3D volumes from a single x-ray. However, these methods often struggle to extrapolate breathing motion or maintain robustness against irregular, out-of-distribution data. Furthermore, by focusing exclusively on static volumetric reconstruction, they overlook temporal motion continuity, a vital source of regularization that could mitigate the ill-posed nature of ultra-sparse sampling.

On-the-fly deformation modelling during beam delivery requires deformable image registration (DIR) to calculate deformation vector fields (DVFs) representing point-wise motion between images (Li *et al* 2010, Zhang *et al* 2013). In particular, by registering baseline scans to live images, DIR enables real-time estimation of targets and/or OARs movement. Traditional optimization-based methods, such as free-form B-splines (Rueckert *et al* 1999), Demons (Pennec *et al* 1999), diffeomorphic transformation (Christensen *et al* 1996, Beg *et al* 2005), are often computationally expensive and slow for real-time clinical applications as they iteratively minimize energy functions. Conversely, AI-driven frameworks like VoxelMorph (U-Net based) (Jaderberg *et al* 2015, Ronneberger *et al* 2015, Balakrishnan *et al* 2019) and recent Transformer-based models (Vaswani *et al* 2017, Chen *et al* 2022, Chen *et al* 2024) offer the fast inference required for real-time applications. However, these learning-based methods remain limited by their requirement for aligned volumetric image pairs, which are difficult to acquire during treatment.

To achieve simultaneous real-time volumetric imaging and deformation modeling, (Shao *et al* 2025) recently introduced DREME. This innovative AI framework integrates motion estimation directly into the CBCT reconstruction process. While this joint optimization approach offers significant advantages, the inherent over-parameterization of simultaneous voxel-wise reconstruction and motion estimation can present challenges regarding convergence stability. Additionally, current evaluations of the framework have focused primarily on volumetric image quality and target localization (via one point represented by center of mass); there remains an opportunity to further validate the model’s performance through comprehensive DVF analysis.

Research suggests that typical respiratory motions, excluding unexpected events like coughing, can be consistently modeled with a set of limited parameters. Low *et al* demonstrated that lung and tumor dynamics can be effectively captured using a five-parameter model (2005). Alternatively, machine learning approaches like principal component analysis (PCA) have shown that two coefficients are often sufficient to represent motion variance, though the optimal number remains patient-specific (Zhang *et al* 2007, Li *et al* 2011). Leveraging the low-degrees-of-freedom characteristics of respiratory motion, we propose LADDER, a Latent Accelerated Diffusion framework for Deformation Estimation enabling Real-time volumetric imaging. The LADDER framework leverages the robust generative capabilities of latent diffusion model (LDM) (Rombach *et al* 2022), focusing on low-dimensional deformation fields rather than high-dimensional images. Specifically, the framework parameterizes patient-specific DVF changes through scaling factors and residual maps, inferred via cross-attention conditioning from x-ray projections. These parameters are then applied to baseline DVF and image to achieve high-fidelity, real-time dynamic volumetric imaging.

As summarized in table 1, the proposed LADDER framework fills in a unique technical niche by addressing the limitation of current motion management strategies. Whereas traditional DIR relies on existing 3D volumes, LADDER parametrizes patient-specific DVF changes from 2D projections. Furthermore, unlike conventional reconstruction methods that necessitate extensive x-ray projections for real-time imaging, LADDER utilizes ultra-sparse projections (1–2 views) as a ‘prompt’ to infer low-dimensional DVF variations. This enables the deformation of a baseline image into a real-time volumetric representation. Critically, whereas existing tracking methods are often limited to geometric tumor centroids, LADDER provides full 3D DVF maps. This capability provides essential information for real-time dose calculation and accumulation, which are not supported by most established methods.

## 2. Methods and materials

### 2.1. LADDER framework

As illustrated in figure 1, the entire LADDER framework has two key components: (1) a deformation network pre-trained that generates patient-specific baseline DVF and (2) an LDM-based DIR model that estimates DVF scaling and residual maps to generate intra-treatment real-time DVF and dynamic volumetric images. At the pre-treatment stage, LADDER adopts VoxelMorph (Balakrishnan *et al* 2019) to generate a patient-specific baseline DVF (ϕ) that describes the tumor and normal tissue displacement between two different respiratory phases from patient’s 4DCT images. In this model, the tumor and surrounding healthy tissues are not tracked as independent moving parts; rather, their spatial relationship is maintained within a unified coordinate system that reflects the anatomical deformation of the patient. During the treatment, LADDER utilizes an LDM (Rombach *et al* 2022) to generate real-time DVFs $\left(\phi_{\text{t}}\right)$ at time t by taking both the patient-specific baseline DVF and real-time x-ray projections $\left(p_{t}\right)$. Specifically, the LDM converts the patient-specific baseline DVF to a lower-dimensional latent representation (z) through a diffusion process. Then the reverse diffusion is proceded with cross-attention transformers, where the latent representation acts as the key and value while x-ray projections act as the query. This cross-attention mechanism conditions the baseline DVF on sparse x-ray projections, essentially enabling the network to learn latent representations of patient-specific DVF and update them with projection-derived anatomical cues. These updated representations then pass through a decoder to estimate DVF scaling and residual maps that later are fused with baseline DVF (ϕ) to yield real-time deformation $\left({\hat{\phi }}_{\text{t}}\right)$. Finally, a spatial transformer (Jaderberg *et al* 2015) applies real-time deformation $\left({\hat{\phi }}_{\text{t}}\right)$ to wrap reference image $x_{i}$ to generate real-time 3D images $\left({\hat{x}}_{\text{t}}\right)$.

### 2.2. LDM

As shown in figure 1(b), in LDM (Rombach *et al* 2022), the encoder encodes (or compresses) the patient-specific baseline DVF, $\phi \in R^{H\times W\times L\times 3}$, into a latent space z=ℰ(ϕ), where $z\in R^{h\times w\times l\times c}$ and the decoder 𝒟 decodes the latent into a low-dimensional scaling map $s_{t}\in R^{H^{\prime }\times W^{\prime }\times L^{\prime }\times 1}$ and residual map $r_{t}\in R^{H^{\prime }\times W^{\prime }\times L^{\prime }\times 3}$. Here the encoder compresses the DVF by a factor $f_{1}=H/h=W/w=L/l=2^{m}$ and down-sample rate of scaling/residual maps $f_{2}=H/h=W/W^{\prime }=L/L^{\prime }=2^{n}$ with m and n are integers. The low-dimensional latent space not only improves computational efficiency but also provides the diffusion model (DM) with important semantic detail for effective generation. In this study, we investigate compression rate $f_{1}$ and down-sample rate $f_{2}$ with m,n=1,2,3,4 on model accuracy and efficiency.

The LDM conditions the DM by integrating a cross-attention mechanism into the U-Net architecture. To harmonize multi-modal conditioning signals (e.g. language prompts, imaging data), the LDM employs a modality-specific encoder (τ) that transforms raw conditioners into a latent embedding space. These embeddings are then adaptively fused with the U-Net’s intermediate feature maps via cross-attention layers, enabling dynamic control over the DM. In this study, we use 2D x-ray projections as a conditioner. Specifically, by axially replicating the ith 2D x-ray projection $\left(p_{t}^{i}\right)$ at the time t across sequential slices, we synthesize a 3D volumetric image $\left(v_{t}^{i}\right)$, effectively extending the acquired 2D data into a pseudo-3D representation. Then the encoder τ converts $v_{t}^{i}$ to an intermediate representation $\tau \left(v_{t}^{i}\right)$ and connected to the U-Net with a cross-attention layer (Q,K,V) with $=W_{Q}\cdot \varphi (z),\text{K}=W_{K}\cdot \tau \left(v_{t}^{i}\right),\text{V}=W_{V}\cdot \tau \left(v_{t}^{i}\right)$. Here φ denotes an intermediate representation of ∈, an estimated noise. $W_{Q},W_{K},W_{V}$ are learnable projection matrices in attention layers. Based on this image-conditioning, the LDM loss can be rewritten as ${loss}_{\text{LDM}}{\left\|\epsilon -\overset{ˆ}{\epsilon }\left(z,\tau \left(v_{t}^{i}\right)\right)\right\|}_{2}^{2}$, where the noise ∈ is determined through LDM (Rombach *et al* 2022).

### 2.3. Loss function of LDM-based DIR

Figure 2 illustrates our physics-informed LDM-based DIR model. In addition to image similarity and DVF smoothness used in conventional DIR, our LDM-based DIR augments the loss with physics-informed constraints, including OAR matching and projection-space similarity terms. OAR matching enforces contour correspondence to precisely align OARs volumes in 3D imaging domain, while projection-similarity metrics evaluate anatomical fidelity in the projection domain (e.g. spine and lungs); both are standard methods for verifying registration accuracy in RT clinical practice. The overall loss function of our LDM-based DIR model consists of four components:

(1) $$L={\text{loss}}_{\text{sim}}+\lambda_{\phi }*{\text{loss}}_{\text{smooth}}+\lambda_{1}*{\text{loss}}_{\text{LDM}}+\lambda_{2}*{\text{loss}}_{\text{phy}}.$$

Each sub-loss component is defined as follows: (1) the image similarity loss ${\text{loss}}_{\text{sim}}$ is defined as ${\text{loss}}_{\text{sim}}=\text{NCC}\left({\hat{\text{x}}}_{\text{t}},{\text{x}}_{\text{t}}\right)$, where a normalized cross-correlation $\text{NCC}\;(\cdot ,\cdot )=\sum_{{\text{x}}_{\text{t}}}\frac{\sum \left({\hat{\text{x}}}_{\text{t}}-\bar{{\hat{\text{x}}}_{\text{t}}}\right)\left({\text{x}}_{\text{t}}-\bar{{\text{x}}_{\text{t}}}\right)}{\sqrt{\sum {\left({\text{x}}_{\text{t}}-\bar{{\text{x}}_{\text{t}}}\right)}^{2}}\sqrt{\sum {\left({\hat{\text{x}}}_{\text{t}}-{\hat{\text{x}}}_{\text{t}}\right)}^{2}}}$ calculates the normalized similarity loss between the warped image $\left({\hat{\text{x}}}_{\text{t}}\right)$ and ground truth $\left({\text{x}}_{\text{t}}\right)$ at time t. (2) The DVF smoothness loss ${\text{loss}}_{\text{smooth}}$ is defined as ${\text{loss}}_{\text{smooth}}={\left\|\nabla {\hat{\phi }}_{\text{t}}\right\|}_{2}^{2}$, where ${\left\|\cdot \right\|}_{2}^{2}$ denotes the L2-Norm operator and ∇⋅ represents the gradient of the DVF. (3) The physics-informed loss ${\text{loss}}_{\text{phy}}$ aligns estimated x-ray projections and segmented OAR masks with their training-data counterparts, respectively: ${\text{loss}}_{\text{phy}}=\;{\text{loss}}_{\text{proj}}+{\text{loss}}_{\text{OAR}}$. The projection-similarity term is given by: ${\text{loss}}_{\text{proj}}=\sum_{i=1}^{I}\text{NCC}\left({\overset{ˆ}{\text{p}}}_{\text{t}}^{\text{i}},{\text{p}}_{\text{t}}^{\text{i}}\right)$ with I ≤ 2 for the existing clinical practice. and the OAR-similarity term is given by: ${\text{loss}}_{\text{OAR}}=\sum_{\text{j}=1}^{\text{J}}\text{DC}\left({\hat{\text{m}}}_{\text{t},j},{\text{m}}_{\text{t},j}\right)$ to ensure anatomical consistency in both projection and image domains. Here, NCC (⋅,⋅) measures the similarity between the warped (deformed) image’s projection $\left({\hat{\text{p}}}_{\text{t}}^{\text{i}}\right)$ and simulated projections $\left({\text{p}}_{\text{t}}^{\text{i}}\right)$ and, denotes the dice coefficient (DC), which quantifies the overlap between registered and ground-truth segmentations across J OARs (where J is the number of OARs). Here, the simulated x-ray projections are generated via the Auto-differentiable Digitally Reconstructed Radiographies (DiffDRR) toolbox (Gopalakrishnan and Golland 2022) and the OARs are segmented using an AI-powered tool, TotalSegmentator (Wasserthal *et al* 2023). The total loss is computed as a weighted sum of the four components, with $\lambda_{\phi },\lambda_{1}$ and $\lambda_{2}$ as regularization weights that balance their relative contribution. In this study, we choose $\lambda_{\phi }=0.1$ and $\lambda_{1}=\lambda_{2}=1$. This combined loss function (equation (1)) is jointly optimized to achieve accurate and physically consistent outcomes.

### 2.4. Model training and testing

The overall LADDER framework was trained in two steps. We first trained the VoxelMorph model with 50 patients in total from Learn2Reg2021 and Learn2Reg2022 (Hering *et al* 2023) to establish an accurate DIR model to generate patient-specific DVF. Then, we trained the LDM-based DIR model using a 5-fold cross-validation approach on a dataset breathing-phase-augmented to 500 samples from original Learn2Reg2021 and Learn2Reg2022 (Hering *et al* 2023). To augment the training dataset, we use the two extreme respiratory phases inhalation $\left(T_{00}\right)$ and exhalation $\left(T_{50}\right)$ dataset, to approximate lung motion over a breathing cycle. A B-spline free-form deformation (Rueckert *et al* 1999) registration is conducted between these two phases to obtain a three-dimensional displacement field. Intermediate phases are then created by linearly scaling this displacement field to produce ten temporal steps, and the corresponding image volumes are warped using linear interpolation. The resulting sequence provides a smooth and consistent representation of respiratory motion for network training. Further, we applied DiffDRR toolbox to generate 2D projections and TotalSegmentator to segment lung regions as physics-informed constrained for model training. With all the data prepared, the training was conducted in two batches, and both spanned 400 epochs. We employed the Adam optimizer with an initial learning rate of 5 × 10^−5^ at a decay rate of 0.98 per epoch. The training process for LDM-based DIR model was terminated at 200 epochs with a converged loss, requiring approximately 19 h in total on a workstation equipped with an NVIDIA RTX 3090 GPU and an Intel(R) Core (TM) i5-14 600 K CPU. The trained LADDER framework was tested on a dataset of 10 cases from publicly available database DIR-Lab (www.dir-lab.com). The average inference time of LDM-DIR model was within 0.2 s per set of x-ray projections.

### 2.5. Evaluation metrics

To ensure the developed LADDER framework satisfying RT application, we employed multiple evaluation metrics to comprehensively assess its performance on registration, imaging, and segmentation predictions. Qualitatively, we assessed the performance by comparing the generated 3D image with ground truth images. Quantitatively, we measured (i) registration accuracy using the mean of target registration error (mTRE), (ii) image quality using structural similarity index measure (SSIM) and normalized mean square error (NMSE) for both 3D volumes and 2D projections, and (iii) OAR segmentation accuracy using the DC. Here, the mTRE is defined as $\text{mTRE}=\frac{1}{K}\sum_{k=1}^{K}\left\|{\overset{‾}{Q}}_{k}^{50}-T_{{\overset{ˆ}{\phi }}_{00\to 50}}{\left(q_{k}^{00}\right)}^{-}\right\|$, with $q_{k}^{00}$ as the kth landmark location on $T_{00}$ reference CT, $q_{k}^{50}$ is the corresponding kth landmark location on $T_{50}$ reference CT, $T_{{\overset{ˆ}{\phi }}_{00\to 50}}$ is the transformation based on the estimated DVF of $T_{00}-T_{50}$, and ‖⋅‖ is the Euclidean distance operator. In total, there are K(=300) landmarks in each case for evaluation. $\text{SSIM}\;=\frac{\left(2\mu_{y}\mu_{\overset{ˆ}{y}}+C_{1}\right)\left(2\sigma_{y\overset{ˆ}{y}}+C_{2}\right)}{\left(\mu_{y}^{2}+\mu_{\overset{ˆ}{y}}^{2}+C_{1}\right)\left(\sigma_{y}^{2}+\sigma_{\overset{ˆ}{y}}^{2}+C_{2}\right)}$, and $\text{NMSE}\;=\frac{\text{y}-{\overset{ˆ}{\text{y}}}_{2}^{2}}{\|\text{y}{\|}_{2}^{2}}$ with y and $\hat{y}$ denoted as the ground truth and the predicted 3D volumetric image (or 2D projection image) respectively. $\mu_{y}$ and $\mu_{\overset{ˆ}{y}}$ are intensity means of images y and $\hat{y},\sigma_{y\overset{ˆ}{y}}$ is the corresponding covariance, $\sigma_{y}^{2}$ and $\sigma_{\overset{ˆ}{y}}^{2}$ are their variances, and $C_{1}$ and $C_{2}$ are small constants to stabilize the division, which are set as 1 × 10^−6^.

## 3. Experiments and results

### 3.1. Configuration parameters selection for LADDER

We evaluated how LADDER configuration parameters (table 2) affect registration accuracy (measured by target registration error (TRE) against ground-truth landmarks), memory usage, and runtime on DIR-Lab dataset. Configuration parameters vary across (i) latent compression rate $f_{1}$ (section 3.1.1), (ii) down-sampling rate $f_{2}$ for scaling/residual maps (section 3.1.2), and (iii) the inhale–exhale span of patient-specific baseline DVFs (section 3.1.3). These studies identified effective $f_{1},f_{2}$ settings, and the inhale-exhale span phases for optimal LADDER performance.

#### 3.1.1. Impact of compression rate $\left(f_{1}\right)$ on DVF accuracy

To understand how latent compression rate influences deformation accuracy and computing speed, we varied $f_{1}$ across 2, 4, 8, and 16, while fixing the decoder down-sampling rate at 8, using dual-projection inputs, and employing a baseline DVF generated on $T_{00}-T_{50}$ phase pair. A moderate level of compression produced the most balanced performance: across the ten DIR-Lab cases, ${\text{f}}_{1}=8$ maintained a low average of mTRE of 0.87 mm across 10 cases while keeping GPU memory usage of 1.22 GB and inferencing time of 0.11 sec (figures 3(a)–(c)). Furthermore, paired t-test demonstrated that differences of TRE between different compression rates (2, 4, and 16 vs 8) are statistically significant in all cases. Lower compression ($f_{1}=2$ or 4) further reduced the mTRE but increased GPU memory usage from 1.22 GB $\left(f_{1}=8\right)$ to 1.52 GB $\left(f_{1}=4\right)$ and 2.01 GB $\left(f_{1}=2\right)$ and inferencing time from 0.11 s $\left(f_{1}=8\right)$ to 0.15 s $\left(f_{1}=4\right)$ or 0.29 s $\left(f_{1}=2\right)$, more than 35.8%. These results indicate that accurate deformation can be achieved without sacrificing real-time feasibility when $f_{1}=8$.

#### 3.1.2. Impact of scaling and residual maps down-sampling rate $\left(f_{2}\right)$

We next examined how the down-sampling rate for the scaling and residual maps affect performance while maintaining the same conditions of encoder compression rate at 8, dual-projection inputs, and a baseline DVF based on $T_{00}$ to phases pair. As shown in figures 4(a)–(c), decreasing $f_{2}$ to value of 2 or 4 improved the mTRE at the expense of GPU memory from 1.19 GB $\left(f_{2}=8\right)$ to 1.50 GB $\left(f_{2}=4\right)$ or 2.01 GB $\left(f_{2}=2\right)$, and runtime from 0.11 s $\left(f_{2}=8\right)$ to 0.15 s $\left(f_{2}=4\right)$ or 0.29 s $\left(f_{2}=2\right)$, while increasing to $f_{2}=8$ accelerated inference with a moderate loss in the average of mTRE across 10 cases from 0.79 mm $\left(f_{2}=2\right)$ to 0.87 mm $\left(f_{2}=8\right)$. Paired t-test demonstrated that differences of TRE between different down-sampling rates (2, 4, and 16 vs 8) are statistically significant in all cases. In practice, $f_{2}=8$ remained stable across patients and phases and provided a practical operating point. Together with results in section 3.1.1, these results suggest setting $\left(f_{1},f_{2}\right)$ to values of (8, 8) to match hardware budgets while preserving deformation fidelity.

#### 3.1.3. Impact of patient-specific baseline DVF

We studied how the inhale–exhale span of the patient-specific baseline DVF affects registration accuracy. In this experiment, the encoder compression rate and decoder down-sampling rate were fixed at 8, and dual-projection inputs were used. We select $T_{00}-T_{10},T_{00}-T_{30}$, and $T_{00}-T_{50}$ as patient-specific baseline DVFs because they represent the critical transition and extreme motion ranges of the respiratory cycle. Phases beyond $T_{50}$ are excluded to avoid redundancy, as the anatomical positions of inspiratory-expiratory transition phases $\left(T_{10},T_{20},T_{30},T_{40}\right)$ often mirror those in the expiratory-inspiratory transition phases $\left(T_{90},T_{80},T_{70},T_{60}\right)$. Thus, the $T_{00}-T_{10},T_{00}-T_{30}$, and $T_{00}-T_{50}$ ranges provide a representative set of displacement vectors spanning small, moderate, and large anatomical deformations, sufficient for robust baseline validation. As shown in figure 5, the LADDER framework effectively leveraged these baseline deformations, stabilizing the scaling to intermediate phases, with the mTRE < 0.94 mm for all cases. And the wider inhale–exhale baselines which capture the broader respiratory motion generally reduced TRE. However, as shown in figure 5, in most cases, paired t-tests ($T_{00}-T_{10}$ and $T_{00}-T_{30}$ vs $T_{00}-T_{50}$) show that the accuracy improvements are not statistically significant. These findings support (i) using baseline DVFs from extreme inhalation-exhalation phases could improve accuracy of subsequent phase inference; (ii) recovering the phase out of baseline DVF respiratory range could be possible.

Collectively, based on the above studies, we determined the optimal configuration of LADDER to be $f_{1}=8,f_{2}=8$, and the $T_{00}-T_{50}$ baseline DVF, which enables a fast inference (around 110 ms) without substantial loss of accuracy. Thus, we will use this set of configurations for the following studies.

### 3.2. The registration accuracy

We evaluated the registration accuracy of the LADDER framework using both single- (anterior–posterior; AP) and dual- [AP and RL (right–left)] projection inputs. These evaluations employed the optimal configuration parameters identified in the preceding analysis and were benchmarked against VoxelMorph 3D–3D registration performed between $T_{00}$ and $T_{50}$ reference CT image pair of 10 validation tests cases. Figure 6(a) compares the TRE values for VoxelMorph and LADDER across both projection configurations. The LADDER framework with dual-projection inputs achieved an average mTRE of 0.87 ± 0.33 mm across 10 cases. This performance was slightly better than the single-projection configuration (0.93 ± 0.34 mm) and the VoxelMorph approach (0.96 ± 0.35 mm). Paired t-tests revealed that the improvement in TRE using dual-projection inputs compared to VoxelMorph was statistically significant across the 10 cases (p<0.05). However, no consistent significant difference was observed between the single- and dual-projection configurations. These results suggest that our DVF parameterization effectively captures the underlying deformation manifold, enabling high-fidelity 3D registration without the computational overhead of traditional 3D-to-3D DIR. We further evaluated the TRE values across five lung lobes and the whole lung, using histograms to visualize the distribution of registration errors by regions. As illustrated, most landmarks exhibited TRE values below 1.0 mm in the whole lung and errors are larger in the upper lobes than in the lower lobes of both lungs.

Although the quantitative mTRE improvement was not substantial, dual-projection inputs provided a more comprehensive 3D reconstruction by resolving motion along anatomical axes that are poorly constrained in a single projection setting. This is qualitatively illustrated in a representative case (Case 2 at phase $T_{30}$) shown in figure 7. Although both single- and dual-projection configurations performed well along the AP direction, the dual-projection approach better resolved deformations along the RL direction, which is poorly constrained in a single-projection setup because of limited depth information. This improvement is evidenced by the smaller image-intensity differences in figures 7(b2) and (b4) compared to those in figures 7(a2) and (a4). These findings suggest that complementary angular information enhances the fidelity of the estimated deformation field. To assess accuracy near the cardiac region, we provided a cross-sectional view through the heart. Due to the inherent visual density of quiver plots, precise qualitative evaluation of registration (motion vector) accuracy in this region remains challenging. However, the image-intensity difference between the ground-truth and the deformed images were larger at the heart boundary.

We further investigated whether increasing the number of projection inputs beyond two could improve registration accuracy, although such configurations are not standard in current clinical practice. As detailed in table 3, the mean and standard deviation of TRE values were evaluated across ten DIR-Lab cases using various projection combinations. Although increasing the number of projections produced in a downward trend in TRE, indicating improved accuracy, the gains were marginal and did not reach statistical significance. This suggests that orthogonal dual-projection inputs provide sufficient anatomical information for LADDER framework to accurately capture complex 3D respiratory motion. Although these results are promising, further validation across a broader range of clinical scenarios is warranted to determine the optimal balance between imaging dose (proportional to the number of projection inputs) and motion-estimation accuracy.

### 3.3. The quality of warped volumetric images

We compared the quality of wrapped volumes against that of ground truth with single- or dual- projection inputs across 9 phases from $T_{10}$ to $T_{90}$ with optimal configuration parameter identified above. From the NMSE results in figure 8(a), we see that both inputs (single- or dual-projections) yield precise warped volumetric images with the mean NMSE < 0.006 across different breathing phases, demonstrating the LADDER framework’s ability for accurate volumetric image generation. Moreover, incorporating an additional projection (in the RL direction) reduces the mean NMSE and stabilizes time series deformation predictions, as evidenced by the lower standard deviation of NMSE compared to using a single-projection input. The SSIM results in panel figure 8(b) exhibit similar trends, with two-projection inputs achieving consistently high structural similarity (SSIM > 0.95).

### 3.4. Physical consistency of warped volumetric images

To verify physical consistency, we compare the warped volume’s DRR (projections) and segmentations against those of the ground-truth volume using single- and dual-projection inputs. Figures 9 and 10 plot bar charts representing mean and std calculated across the 9 breathing phases $\left(T_{10}-T_{90}\right)$ for each specific patient-level case. Compared to the single-projection input, the dual-projection inputs increased SSIM for the warped images’ DRRs along both views across all cases of DIR-Lab dataset. With dual-projection inputs, similar SSIM accuracy of the warped images’ AP DRRs observed, whereas they improved substantially and exhibited smaller variability of the generated RL projections (figure 9). Segmentation accuracy on warped images followed the same trend (figure 10), higher accuracy with dual-projection inputs compared to single-projection inputs. These results demonstrate that leveraging the dual-projection enhances structural fidelity, thereby underscoring the benefit of dual-projection information in continuous deformation. Across these twelve organs, the whole lung and lung lobes achieve high segmentation accuracy (mean DC > 0.98) with both single- and dual-projection inputs. The segmentation accuracy of trachea, bronchia, and pulmonary airway are still high (mean DC > 0.85), though lower than lungs. It is observed that organs belong to cardiac vascular system, including heart and pulmonary artery have the lowest accuracy and largest variation between cases. These discrepancies are due to the physics-informed model being primarily trained using lung segmentation as the physical guidance.

## 4. Discussion

The LADDER framework establishes a paradigm for real-time 3D deformation and volumetric imaging estimation by combining a patient-specific prior with an LDM conditioned on sparse x-ray projections. By restricting deformation estimation to a low-dimensional DVF manifold and updating deformation through learned scaling and residual components, the framework addresses the ill-posed nature of reconstructing 3D deformation from ultra-sparse 2D imaging. The experimental results demonstrate that this design enables accurate and computationally efficient deformation estimation under clinically relevant imaging constraints.

The proposed LADDER framework is intended to integrate into the lung SBRT (both photon and proton) workflow as follows: (1) Pre-Treatment Phase (Simulation & Planning): A standard 4DCT is acquired during simulation to delineate the planning target and OARs, support treatment planning, and characterize the respiratory motion for establishing the patient-specific baseline DVFs. (2) Treatment Setup & Verification: The patient is positioned using room lasers and immobilization devices. A pretreatment 3D or 4D CBCT scan is then performed for initial volumetric verification and couch-shift correction. (3) Treatment Delivery & Tracking: By leveraging the continuous stream of 2D x-ray projections acquired during treatment delivery, LADDER enables real-time processing and generates instantaneous 3D images. The target can then be automatically segmented. The generated real-time 3D image, together with the segmented target can be used for the subsequent real-time motion management strategy, monitoring/tracking (Bertholet *et al* 2019), and adaptive plan optimization (Lu *et al* 2009)

The performance of our system compares favorably with that reported for existing 3D-based methods. Conventional 3D CBCT, the current standard for SBRT setup verification, produces images that are inherently degraded by respiratory motion artifacts, resulting in blurred tumor representations that limit achievable registration accuracy regardless of the algorithm used. Our method explicitly models respiratory motion through a patient-specific learned deformation framework, LADDER. This framework estimates 3D deformation fields from sparse intrafraction x-ray projections by constraining estimation to a low-dimensional DVF manifold, thereby addressing the ill-posed problem of reconstructing 3D motion from ultra-sparse 2D imaging without relying on motion-corrupted volumetric data. Using this approach we achieved an average mTRE of 0.87 mm across 10 test cases, which is well within the AAPM TG-142 tolerance for SBRT (⩽1 mm) (Klein *et al* 2009). We also demonstrate improved registration accuracy relative to VoxelMorph 3D-3D registration on the same dataset (figure 6(a)). Furthermore, conventional 3D methods typically rely on an ITV with 5–10 mm margins to compensate for motion. By providing real-time volumetric data, our system facilitates a transition from these broad ‘motion envelopes’ to precise tracking. This capability may enable reduced planning margins, potentially spare healthy lung tissue and lower the risk of treatment-induced toxicity without sacrificing localization accuracy. From clinical workflow perspective, our method uses projections acquired during treatment, potentially reducing pretreatment procedure time by bypassing the need for a dedicated CBCT scan. The cumulative radiation dose is directly proportional to the number of projections acquired. By adopting existing clinical protocols, such as the CyberKnife real-time imaging acquisition frequency, the system may balance between high-frequency tracking with patient safety while maintaining radiation exposure within clinically approved limits.

A primary challenge in 2D projection-based tracking is the inherent loss of depth information. In this work, we overcome this limit not through traditional geometric back-projection, but by leveraging a patient-specific DIR prior integrated with an LDM. Unlike traditional dimensionality-reduction techniques that may discard depth information, LADDER uses 2D projections as conditional cues to navigate a high-dimensional, patient-specific 3D latent space. By integrating a DIR-based motion prior with an LDM, the system does not simply reduce dimensionality. This generative approach allows the system to ‘recover’ depth by constraining the reconstruction within a biologically plausible motion manifold derived from the patient’s own anatomical data. Consequently, the model achieves accurate registration by identifying the optimal 3D state consistent with the observed 2D projections. By tracking global 3D motion rather than isolated tumor movement, the system accounts for the complex interplay between the target and adjacent OARs, which is particularly important for accurate dose evaluation in beampath-critical therapies such as proton and heavy-ion RT.

While digital and physical phantoms provide a known ‘Ground Truth’ for initial benchmarking, they often lack the anatomical complexity and stochastic nature of human physiology. Phantoms typically rely on simplified geometry and predictable, linear motion patterns that do not fully capture the non-linear respiratory deformations encountered in a clinical setting (Castillo *et al* 2009). We posit that for DIR-based applications, validation using actual patient image data is a more rigorous test of spatial accuracy. By validating our framework against 4DCT datasets containing 300 expert-validated landmarks per case, we ensure that the reported precision reflects real-world performance rather than the idealized conditions of a phantom. The highly non-linear nature of respiratory motion presents a significant risk for motion underestimation, particularly in single-projection systems. To mitigate this, our framework utilizes orthogonal views (AP and RL), which provide the primary axes of motion simultaneously (Keall *et al* 2006). Furthermore, the combination of DIR and LDM is specifically designed to model non-linear intensity changes rather than assuming rigid or linear trajectories. This architecture supports that complex, patient-specific deformations are captured and reconstructed accurately, providing a reliable estimation of 3D motion even during irregular breathing cycles.

A potential concern in dual-projection reconstruction is the temporal gap required for gantry rotation, which could introduce errors due to intra-fractional motions. Our framework assumes simultaneous acquisition, as available on clinical IGRT platforms such as the CyberKnife robotic radiosurgery system (Accuray Inc., Sunnyvale, CA) with orthogonal kV imaging (Kilby *et al* 2010) and the ExacTrac system (BrainLab AG, Munich, Germany) with dual stereoscopic x-ray imaging (Muacevic *et al* 2006, Jin *et al* 2008). Furthermore, the generative nature of the LDM allows 2D inputs to be mapped onto a 3D latent manifold, helping to prevent reconstruction artifacts or motion underestimation during high-frequency motion. A great challenge in 2D-based motion management is the presence of ‘blind spots’ where the target is obscured by dense anatomical structures, such as the ribs or the heart (Gefter *et al* 2023). Our LADDER framework addresses this through a global feature-learning approach rather than local target tracking. By employing an LDM that considers the entire projection intensity map, the system can infer 3D tumor position from secondary anatomical indicators and deformation patterns, even when the primary target is partially obscured.

Using dual-projection input, compression and down-sampling rate of 8, and an end-inhale to end-exhale baseline DVF, LADDER achieves an mTRE of 0.87 ± 0.33 mm across 10 test cases, together with high volumetric structure similarity (3D SSIM > 0.95) and low reconstruction error (NMSE < 0.006), while maintaining real-time inference (~0.11–0.12 s). These results indicate that, when appropriate compression and down-sampling rates are selected, the proposed framework can recover accurate deformation from sparse projection data while meeting the computational latency requirements for online tracking. The influence of configuration parameter selection clarifies the balance between accuracy and efficiency (figure 3). Analysis of the compression rate $\left(f_{1}\right)$ shows that reducing compression leads to modest improvements in TRE but requires substantially higher memory usage and longer inference time. In contrast, moderate compression $\left(f_{1}=8\right)$ preserves most of the deformation accuracy while significantly improving computational efficiency. These results suggest that the latent representation captures dominant respiratory motion characteristics even under compression, which is advantageous for real-time deployment. However, the measurable TRE differences also indicate that excessive compression reduces sensitivity to fine-scale deformation, highlighting a trade-off between accuracy and resource use. A similar accuracy–efficiency relationship is observed when varying the down-sampling rate of the scaling and residual maps $\left(f_{2}\right)$ as shown in figure 4. Lower down-sampling yields slightly improved deformation accuracy, whereas higher down-sampling reduces memory usage and inference time with only a limited increase in TRE. Importantly, accuracy degradation occurs gradually rather than abruptly from $f_{2}=2-8$, indicating stable behavior across a range of decoder resolutions. Although we selected compression rate and down-sampling rate of 8 based on our hardware condition, these parameter studies demonstrate that LADDER can be adapted to different computational environments. Notably, even under aggressive compression and down-sampling, the framework preserves reasonable deformation accuracy.

The analysis of patient-specific baseline DVF selection provides further insight into the stability of the proposed framework (figure 5). Baselines constructed from end-inhale to end-exhale phases yield the best overall accuracy. However, the results show that LADDER remains effective across different baseline spans, with an average mTRE across 10 cases decreasing from approximately 0.94 mm to 0.87 mm as the baseline span increases (figure 5). This small range of variation indicates stable performance under imperfect baseline selection rather than abrupt degradation. The scalar-based deformation update formulation is consistent with the observed ability to track respiratory motion without requiring precise identification of inhale–exhale endpoint pairs. While optimal baseline selection improves accuracy, the results suggest that acceptable deformation estimation can be achieved when ideal endpoints are unavailable. While using pre-treatment scans to generate patient-specific baseline DVFs is technically sound, real-world clinical challenges remain, specifically intra-fraction motion variability like changing respiratory depth and frequency. Addressing these will require further clinical data and exploration of diverse breathing patterns.

Figures 6 and 7 show the comparison between single-projection and dual-projection conditioning highlights the contribution of real-time geometric (projection) information to deformation estimation accuracy. Dual orthogonal projections consistently reduce mTRE by approximately 0.05 mm relative to single-projection input. This improvement is accompanied by higher structural similarity in the RL direction (see figure 9), as reflected by increased SSIM values than those via single-projection, where deformation is weakly constrained under single-projection geometry. These results indicate that dual-projection conditioning improves directional accuracy rather than merely reducing global error. The primary limitation of dual-projection conditioning is increased acquisition time and/or system complexity, which may constrain its applicability on certain RT machines.

To contextualize the baseline respiratory deformation of the validation cohort prior to registration, the voxel-wise DVF magnitudes between $T_{00}$ and $T_{50}$ phases were evaluated. Table 4 summarizes the mean and absolute ranges (minimum–maximum) of the overall and directional (SI, AP, LR) lung voxel motions for each of the 10 test cases. In contrast to the tumor-specific data summarized in table 2 of the AAPM Report No. 91 (Keall *et al* 2006), the values presented here reflect a whole-organ analysis involving hundreds of thousands of voxels per patient. This comprehensive evaluation captures the full spatial gradient of respiration: voxels localized near the lung apex exhibit minimal to near-zero motion, whereas voxels situated near the diaphragm display pronounced displacements, reaching maximum limits between 12.10 mm and 27.65 mm across the cohort. Globally, the mean lung voxel motion resides on the order of a few millimeters per case, aligning with the shallow-to-normal breathing characteristics outlined in table 3 of the AAPM Report No. 91 (Keall *et al* 2006).

While the specific clinical respiratory protocols (e.g. active motion mitigation strategies) of this public dataset are not explicitly documented, this wide range of baseline motion provides a rigorous test for the registration framework. Despite maximum baseline displacements exceeding 20 mm in multiple cases (e.g. Cases 2, 6, 7, 8 and 10), the proposed registration model successfully accommodated these large, localized deformations, minimizing the maximum TREs at landmarks to ~2 mm (figure 6). Notably, these landmarks (300 points/case) comprise only a small subset voxels within the entire lung, yet they accurately reflect the whole lung motion displacement (Kadoya *et al* 2016). This robust performance highlights the model’s potential clinical utility in managing realistic free-breathing variations without strictly requiring prior physical motion mitigation, though future validation in documented deep-breathing cohorts is warranted.

Evaluation of volumetric image quality and physical consistency further supports the reliability of the proposed approach (figures 8–10). The warped volumes exhibit low NMSE and high projection-space SSIM, indicating strong agreement with measured projection data. Lung volume segmentation achieves consistently high Dice scores across respiratory phases, whereas heart segmentation shows lower accuracy but improves with the dual-projection inputs. The decreased accuracy of heart volumetric information is consistent with its smaller size and more complex motion of the heart, as well as the emphasis on lung anatomy during training. These results also imply that LADDER generalizes well to respiration-dominant structures, while performance for more complex organs may benefit from additional organ-specific constraints. Here, respiration-dominant structures refer to those organs whose geometric position is significantly altered by the respiratory cycle, including the lungs, liver, and esophagus. While our model demonstrates high registration accuracy for these regions, it is important to note that this study does not directly evaluate clinical outcomes. Instead, it provides the underlying motion estimation framework necessary for motion-compensated systems.

Despite these promising results, several limitations remain. First, the 3D anatomical contours used for validation were generated using TotalSegmentator without manual expert refinement. While manual adjustment is often considered the gold standard, it is increasingly recognized as time-prohibitive for large-scale datasets and prone to significant inter-observer variability (Joskowicz *et al* 2019). TotalSegmentator has demonstrated state-of-the-art segmentation performance for thoracic organs, with reported DC of 0.95–0.98 against expert manual contours (Wasserthal *et al* 2023, Hinck *et al* 2025). This level of accuracy justifies its use as a robust and reproducible pseudo-ground truth for volumetric evaluation in this study. Second, we observed lower segmentation accuracy for the heart compared to the lungs. This discrepancy is likely twofold: (1) the model’s training objective prioritized lung anatomy through a lung-volume matching constraint, and (2) the inherent temporal limitations of 4DCT imaging. While 4DCT effectively captures respiratory motion, cardiac motion occurs at a much higher frequency and follows a more complex trajectory that is not typically resolved by respiratory-gated protocols (Roujol *et al* 2013, Ouyang *et al* 2020). Consequently, cardiac boundaries in the reference data may exhibit motion blurring or phase-mismatch artifacts, which naturally limit the reconstructive accuracy for this specific organ. While dual-projection input improves effectiveness, heart and pulmonary artery deformation estimation remains more challenging [mentioned in section 4.3] due to lacking sufficient physical guidance in training and its complex and partially decoupled dynamics from lung motion. This phenomenon indicates opportunities for organ-specific modeling or adaptive loss weighting, and it is beneficial to include specific target organs during the training process. Future work that integrates multi-gated or high-temporal-resolution imaging could further improve cardiac modeling within our framework. Third, the achieved mTRE on the DIR-Lab dataset is particularly notable as it approaches the intrinsic landmark uncertainty of the dataset. (Castillo *et al* 2009) reported mean inter-observer landmark identification errors of 0.74–1.14 mm across five cases, establishing the effective spatial resolution floor of the reference standard. Our average mTRE of 0.87 mm across 10 cases falls within this inter-observer variability range, indicating near-ceiling performance relative to the validation dataset’s measurement precision and suggesting that more precisely annotated datasets could reveal even higher registration accuracy. Also, in this study, we utilized expert-validated anatomical landmarks to quantify registration accuracy, as the utilized public datasets (Learn2Reg and DIR-Lab) do not provide specific tumor contours. While these landmarks serve as a reliable proxy for localized tissue motion, we recognize that explicit tracking of a tumor region is essential for clinical adoption. Our framework is architecturally designed to propagate tumor masks through the reconstructed 3D DVFs, and future studies will focus on evaluating the positional accuracy of tumor in a clinical cohort with known pulmonary lesion location. Fourth, we trained and validated our model using widely recognized benchmarks, the Learn2Reg and DIR-Lab datasets. The Learn2Reg cases represent patients with confirmed lung malignancies and chronic obstructive pulmonary disease with varying degrees of lung inflation, and the DIR-Lab cases consist of thoracic 4DCT scans from esophageal and lung cancer patients, featuring varying degrees of respiratory motion and tumor locations. We acknowledge that these public cohorts do not capture the full spectrum of patient diversity found in broader clinical practice and lack detailed patient-level information (e.g. age, ethnicity, socioeconomic status, tumor location, and specific respiratory patterns), which limits our ability to evaluate potential algorithmic bias; consequently, further validation on more diverse, multi-ethnic datasets is necessary to ensure broader clinical generalizability. Other envisioned future work includes extending LADDER to adaptive baseline updating between treatment fractionation, integrating additional real-time cues (surface, electromagnetic, or integrated kV/MV data), and evaluating dosimetric impacts in online RT scenarios. These directions will help translate LADDER from an accurate algorithmic framework to a dependable clinical tool for real-time motion management.

## 5. Conclusion

In this study, we introduced LADDER, an AI-driven framework that integrates VoxelMorph-based DIR, LDM, and physics-informed constraints to accurately estimate respiratory deformation and reconstruct dynamic volumetric lung images. Leveraging patient-specific baseline DVFs, LADDER enables real-time 3D motion estimation and volumetric reconstruction from ultra-sparse x-ray projections. Across experiments, LADDER consistently achieved sub-millimeter target registration error with real-time inference, demonstrating strong potential for next-generation motion management and image guidance. These capabilities support on-the-fly anatomical modeling and may enhance the safety and efficacy of lung SBRT.

## Figures and Tables

**Figure 1.**
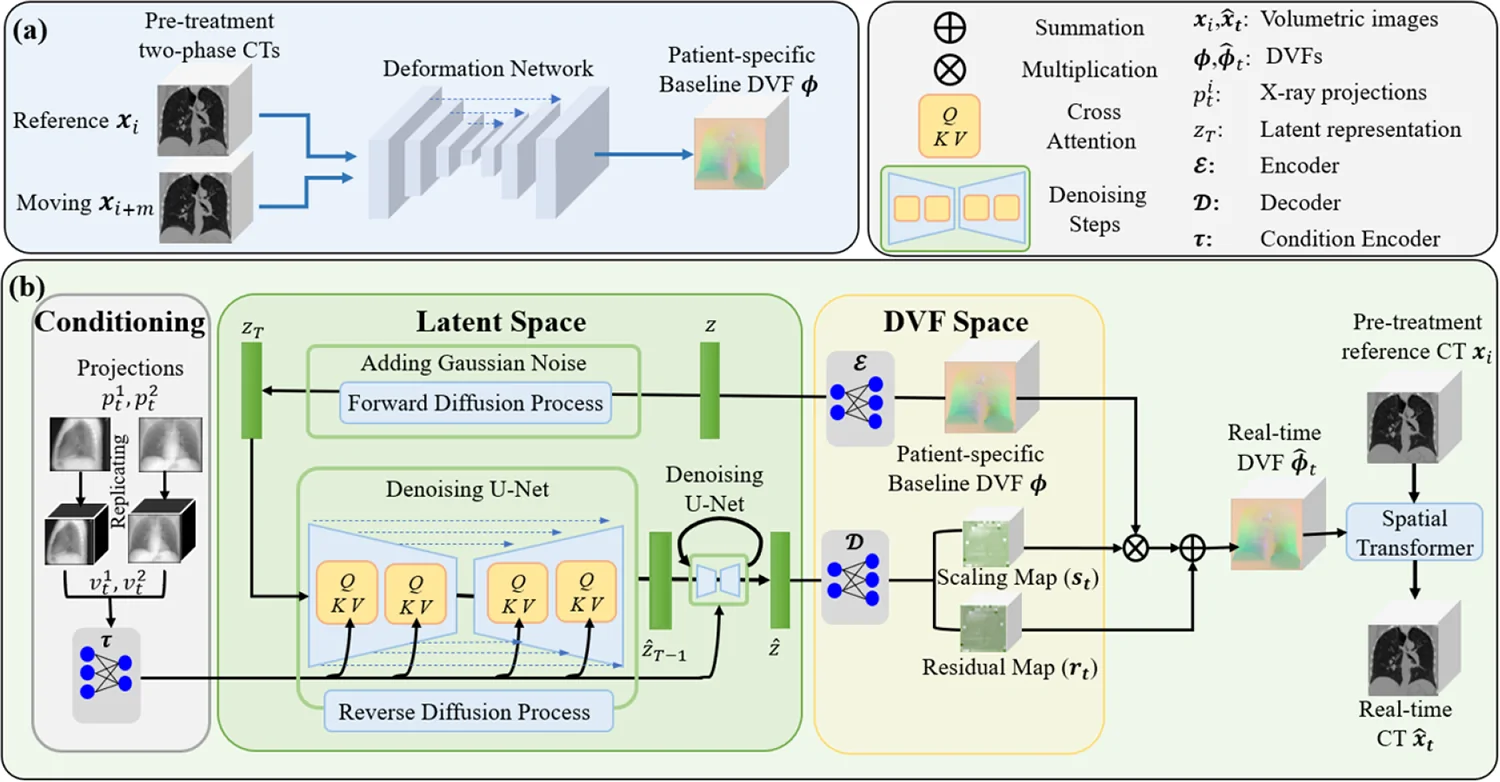
Overview of the LADDER framework. (a) The deformation network initially computes a patient-specific baseline deformation vector field (DVF) from two phases of pre-treatment 4D-CT images. (b) The baseline DVF is used as input to the latent diffusion model (LDM), incorporating x-ray projections via cross-attention to generate low-dimensional scaling and residual maps to capture real-time DVF changes. The full-resolution real-time DVF is yielded by fusing scaling and residual maps with baseline DVF, then used to warp a pre-treatment CT image to produce real-time 3D images.

**Figure 2.**
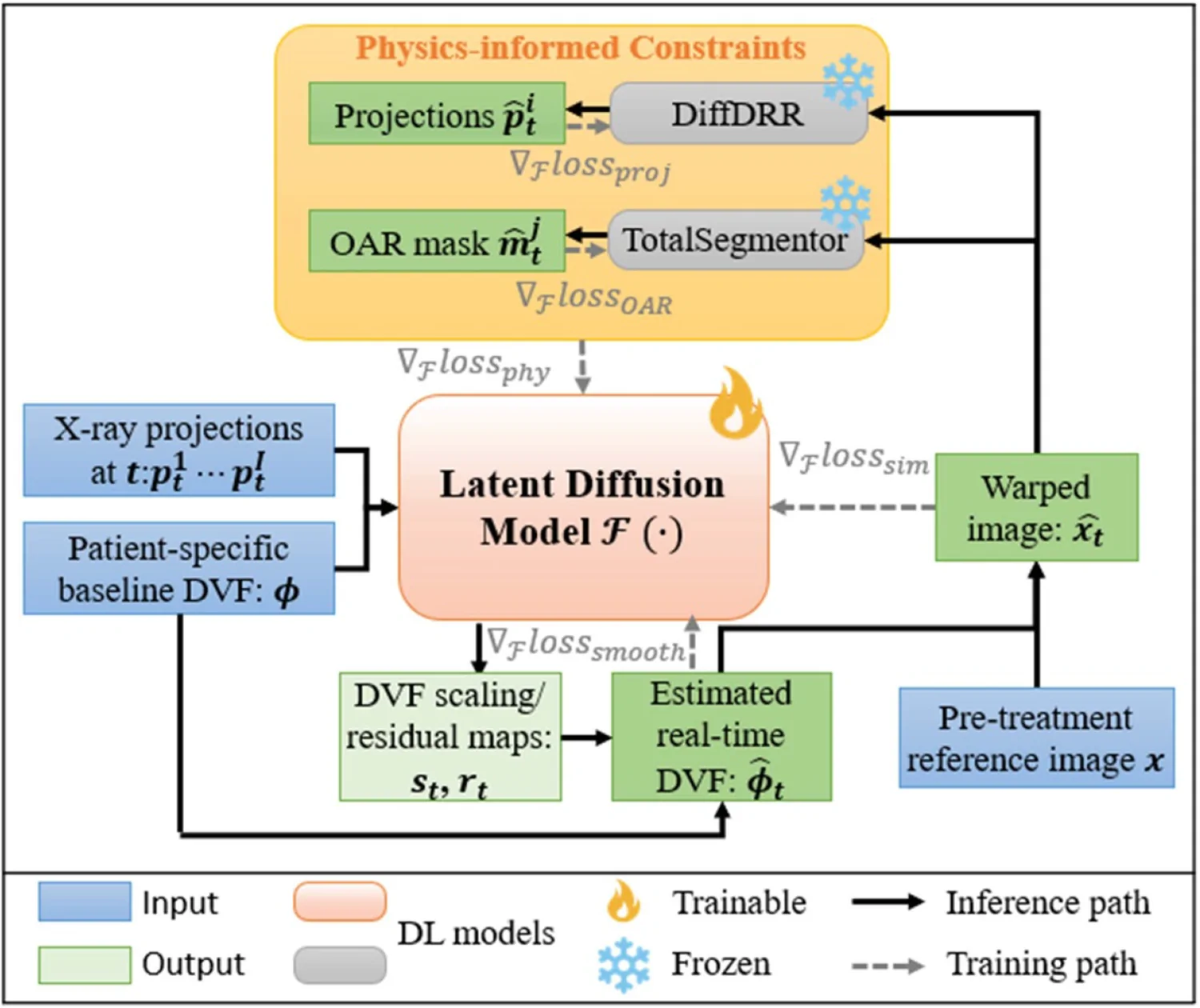
Physics-informed LDM-based DIR model denoted as ℱ(⋅). Inputs (blue): real-time x-ray projections, patient-specific baseline DVF, pre-treatment reference 3D image. Output (light green): DVF scaling and residual maps. Indirect outputs (green): estimated real-time DVF, warped real-time 3D images, projections and OAR masks. The gray dashed arrows depict which components each loss function corresponds to. The operator $\nabla_{ℱ}$ denotes the gradient update for the trainable model ℱ(⋅).

**Figure 3.**
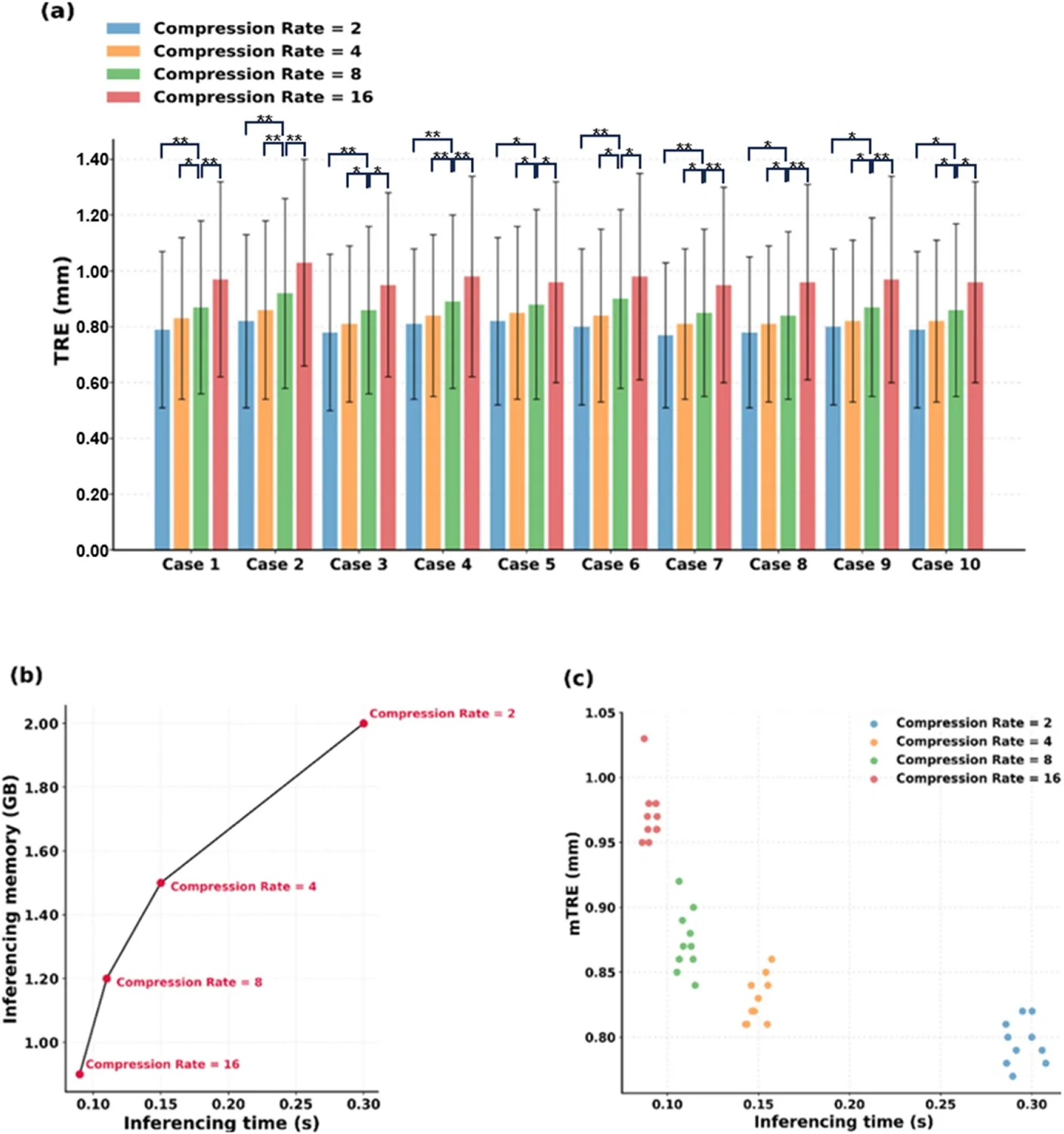
LADDER performance across various cases of compression rate. (a) Bar charts display the mean and standard deviation (std) of TRE in ten DIR-Lab samples under different encoding compression rates of 2, 4, 8 and 16; (b) Inferencing memory versus inferencing time under different compression rates; (c) mTRE versus inferencing time under different compression rates. *:*P* < 0.05 and **:*P* < 0.01for statistical significance between conditions assessed by paired t-test.

**Figure 4.**
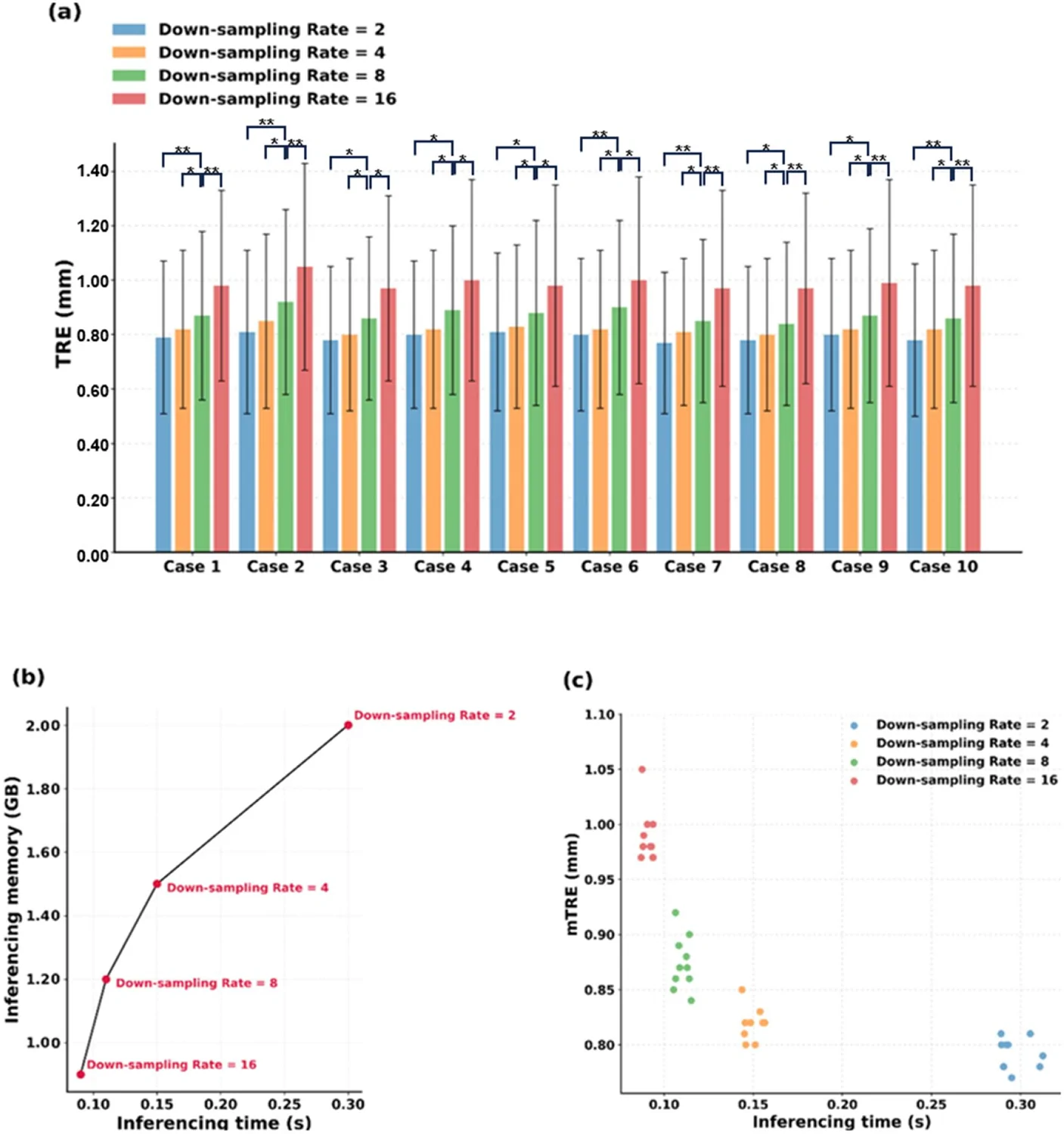
LADDER performance across various cases of scaling and residual maps down-sampling rate. (a) Bar charts display the mean and std of TREs in ten DIR-Lab cases under different down-sampling rates of 2, 4, 8 and 16; (b) Inferencing memory versus inferencing time under different down-sampling rates; (c) mTRE versus inferencing time under different down-sampling rates. *: *P* < 0.05, and **: *P* < 0.01 for statistical significance between conditions assessed by paired t-test.

**Figure 5.**
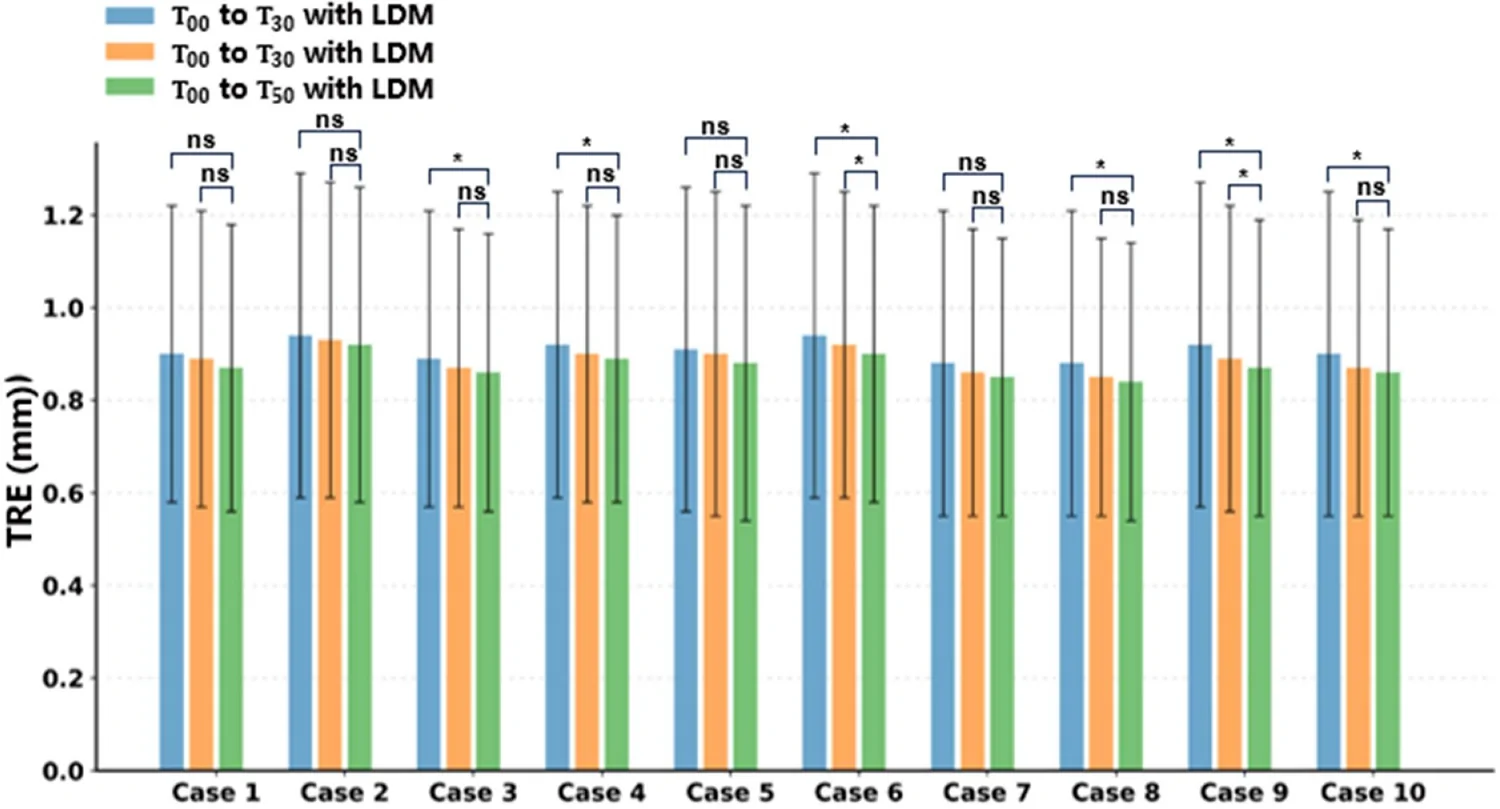
Bar charts display the mean and standard deviation of TRE across breathing-phase spans for patient-specific baseline DVFs in 10 DIR-Lab cases. ns: not significant, and *: *P* < 0.05 for statistical significance between conditions assessed by t-test.

**Figure 6.**
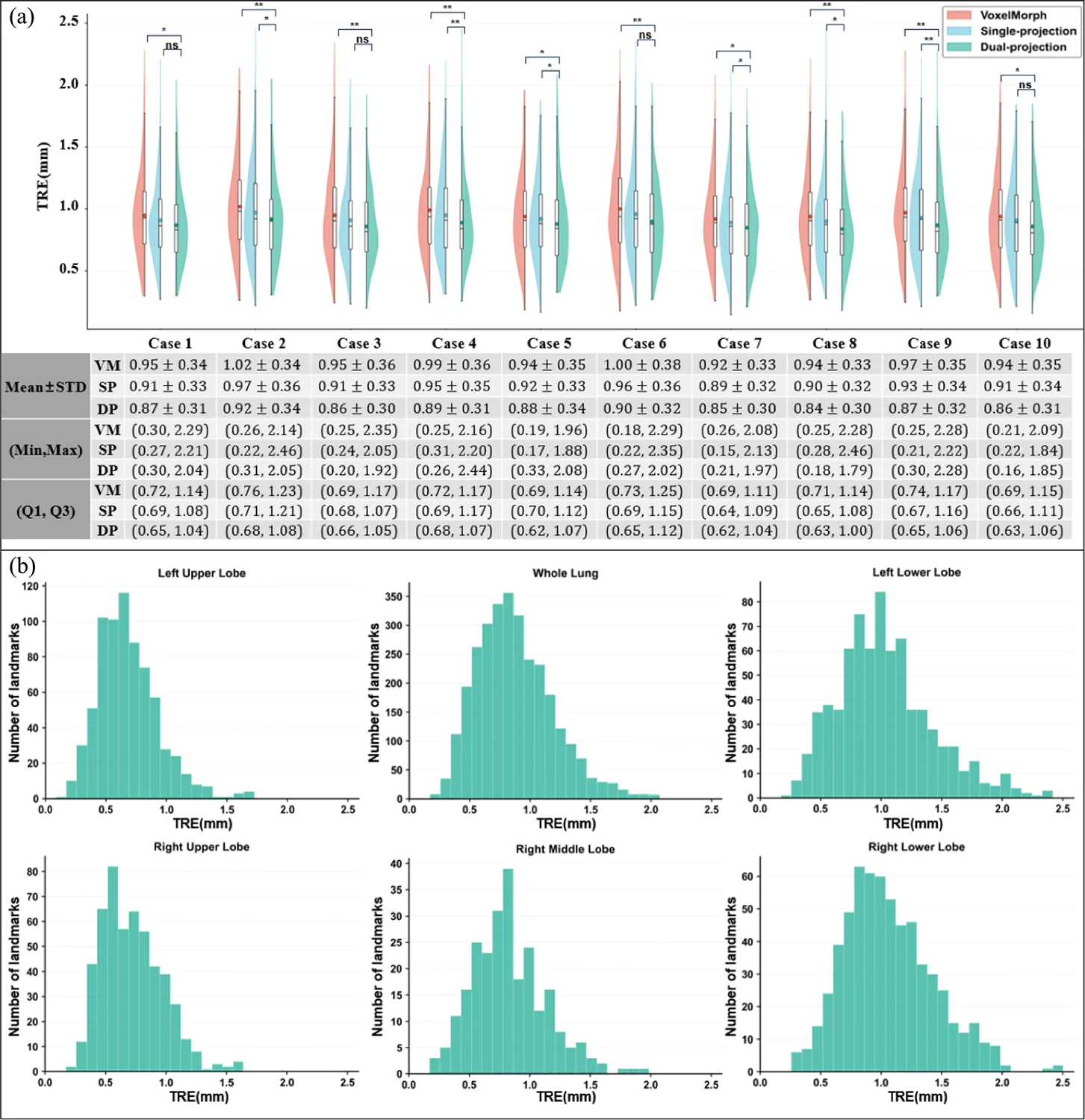
TRE evaluation of 10 cases in DIR-Lab dataset. (a) Violin plots display the TRE (mm) distribution with VoxelMorph (baseline DVFs generated on $T_{00}$ and $T_{50}$ phase pairs), single-projection or dual-projection inputs in 10 DIR-Lab cases (VM: VoxelMorph, SP: single-projection, DP: dual-projection, mean: mean value of TRE, STD: standard deviation, min: minimum error, max: maximum error and Q1/Q3: 1st/3rd quartile) in millimeter. ns: not significant, *: *P* < 0.05, and ** *P* < 0.01 for statistical significance between conditions assessed by t-test. (b) Six histograms of TRE values in 5 lung lobes and the whole lung across 10 DIR-Lab cases.

**Figure 7.**
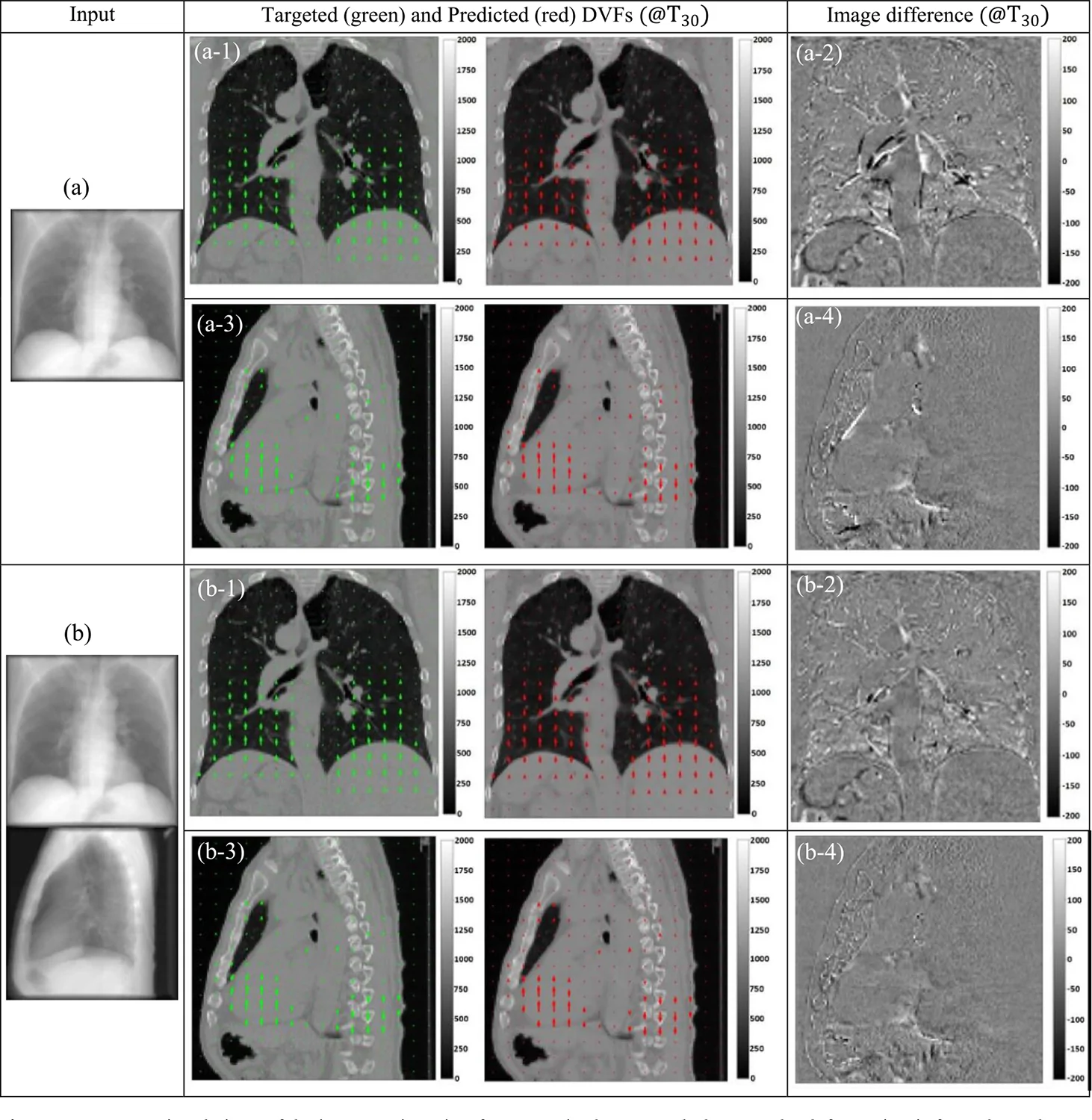
Cross-sectional views of the image registration for Case 2 in the DIR-Lab dataset. The deformation is from the end-of-inhale phase $T_{00}$ to a mid-exhale phase $T_{30}$. (a) and (b) x-ray projection inputs to LADDER. (a-1) and (a-3) Quiver plots of the targeted DVFs calculated using VoxelMorph deformable registration vs the corresponding DVFs predicted by LADDER in coronal and sagittal views. Green arrows represent the targeted DVF amplitudes, and red arrows represent the predicted DVFs. (a-2) and (a-4) Image-intensity differences between the ground-truth and deformed images, highlighting areas of discrepancy. (b-1) and (b-3) and (b-2) and (b-4) show the same analysis as in (a) but using dual AP-RL projection inputs.

**Figure 8.**
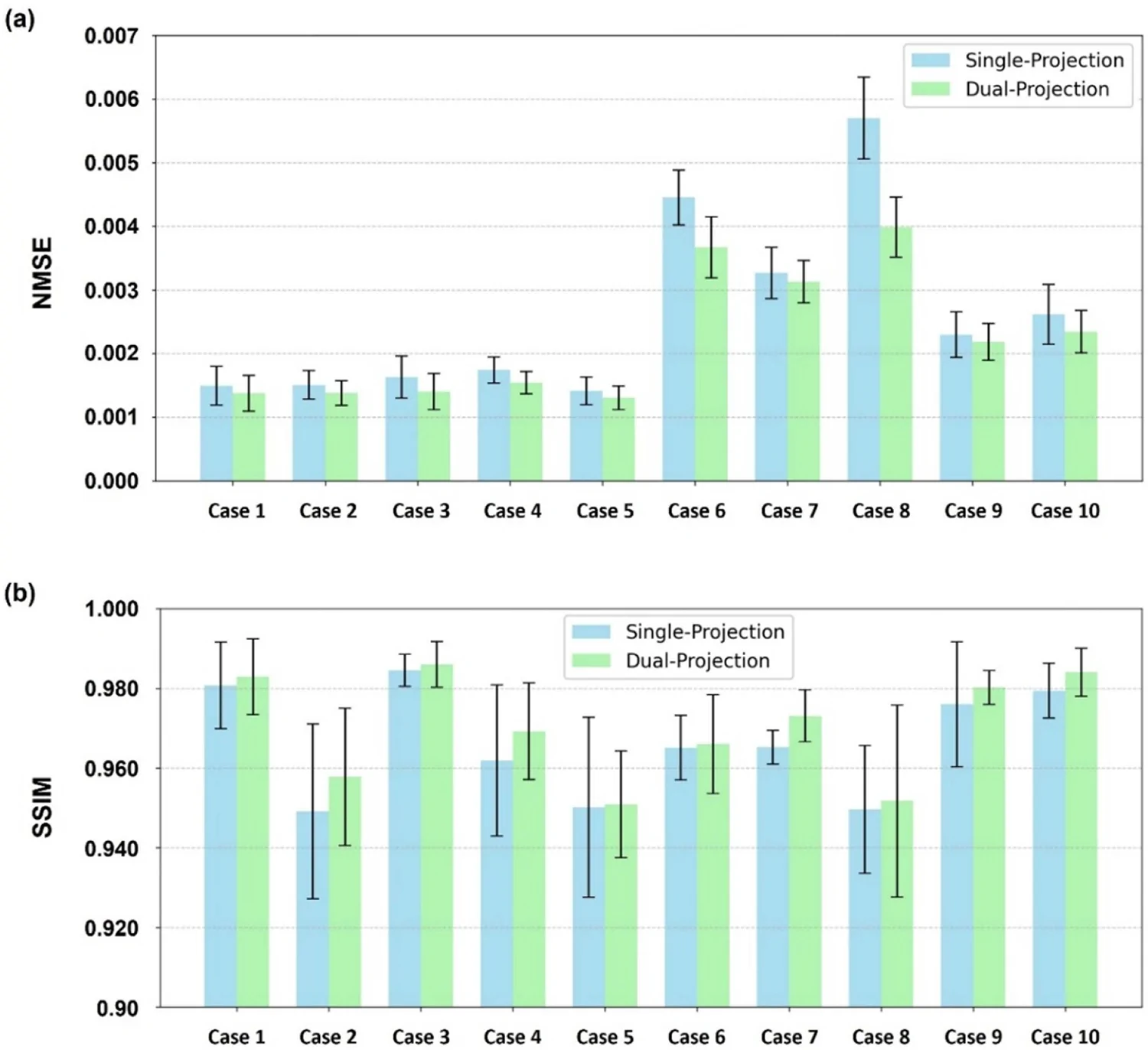
Evaluation of 3D warped CTs at different breathing phases obtained from single- and dual- projections quantified by (a) NMSE and (b) SSIM. The data comprises of 10 cases from the DIR-Lab dataset, with bar charts displaying mean and std calculated across the 9 breathing phases $\left(T_{10}-T_{90}\right)$ for each specific patient-level case.

**Figure 9.**
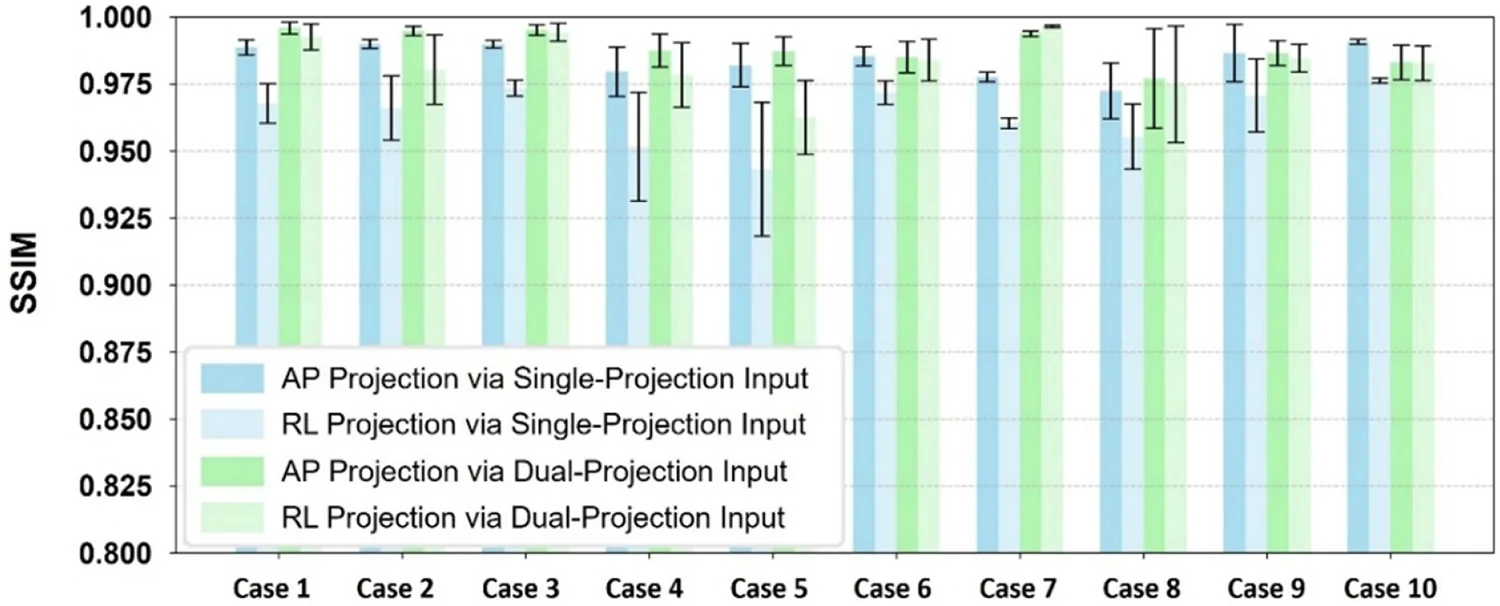
Comparison of SSIM of the generated AP and RL DRRs obtained from single- and dual-projection inputs. The data comprises of 10 cases from the DIR-Lab dataset, with bar charts displaying mean and std of Dice coefficient calculated across the 9 breathing phases $\left(T_{10}-T_{90}\right)$ for each specific patient-level case.

**Figure 10.**
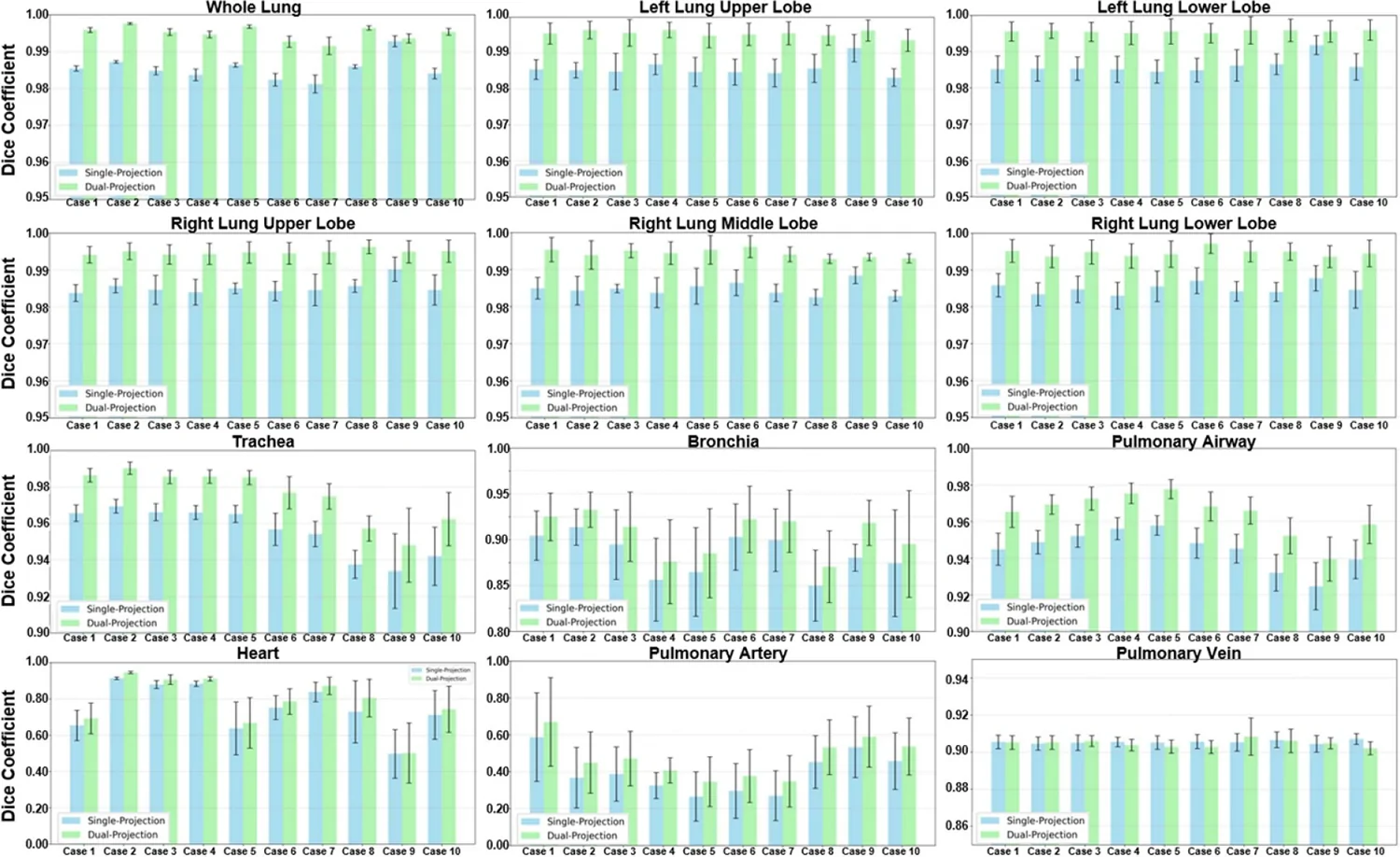
Comparison of OAR segmentation accuracy for lungs, left lung upper lobe, left lung lower lobe, right lung upper lobe, right lung middle lobe, right lung lower lobe, trachea, bronchia, pulmonary airway, heart, pulmonary artery, and pulmonary vein obtained from single- and dual-projection inputs. OAR segmentations on warped images and the reference 4DCT images were all generated by TotalSegmentator. The data comprises of 10 cases from the DIR-Lab dataset, with bar charts displaying mean and std of Dice coefficient calculated across the 9 breathing phases $\left(T_{10}-T_{90}\right)$ for each specific patient-level case.

**Table 1.** Qualitative comparison of the proposed framework against existing techniques. While conventional DIR provides high volumetric accuracy, it cannot operate in real time. Conversely, existing geometric tracking methods offer real-time speed but lack the volumetric deformation data (DVF) necessary for precise dosimetric assessment. The proposed method bridges this gap by providing real-time DVF and imaging from ultra-sparse views.

Feature	Conventional 3D image-based DIR	Sparse-view reconstruction	Real-time tumor tracking	Proposed LADDER framework
Input requirements	3D images	~10–50 projections	2 projections	1–2 projections
Processing latency	High (minutes)	Moderate/high	Low (real-time)	Low (Real-time)
Tracking output	3D DVF	Static 3D volume	3D Centroid (geometric)	3D DVF
Volumetric imaging	Yes (static)	Yes (real-time)	No	Yes (real-time)
Dosimetric utility	High (offline)	Moderate	Low (no density map)	High (real-time)
Ultra-sparse views	No	Yes	Yes	Yes

**Table 2.** Configuration parameters of LADDER.

Name of hyper-parameters	Value
Encoder compression rate	*f*_1_ = 2, 4, 8, 16
Decoder down-sampling rate	*f*_2_ = 2, 4, 8, 16
Patient-specific baseline DVF	*T*_00_–*T*_10_, *T*_00_–*T*_30_, *T*_00_–*T*_50_

**Table 3.** Evaluation of registration accuracy on 10 DIR-Lab cases. Mean and std of TRE (mm) with different projection inputs: AP (0°), RL (90°), ARO: anterior-right oblique (45°); ALO: anterior-left oblique (315°).

Projection inputs	Case 1	Case 2	Case 3	Case 4	Case 5	Case 6	Case 7	Case 8	Case 9	Case 10
AP	0.91 ± 0.33	0.97 ± 0.36	0.91 ± 0.33	0.95 ± 0.35	0.92 ± 0.33	0.96 ± 0.36	0.89 ± 0.32	0.90 ± 0.32	0.93 ± 0.34	0.91 ± 0.34
AP + RL	0.87 ± 0.31	0.92 ± 0.34	0.86 ± 0.30	0.89 ± 0.31	0.88 ± 0.34	0.90 ± 0.32	0.85 ± 0.30	0.84 ± 0.30	0.87 ± 0.32	0.86 ± 0.31
ARO + ALO	0.87 ± 0.31	0.93 ± 0.34	0.87 ± 0.30	0.89 ± 0.31	0.89 ± 0.35	0.91 ± 0.32	0.85 ± 0.31	0.85 ± 0.31	0.88 ± 0.32	0.87 ± 0.31
AP + RL + ARO	0.85 ± 0.30	0.90 ± 0.32	0.84 ± 0.29	0.86 ± 0.31	0.87 ± 0.32	0.87 ± 0.30	0.83 ± 0.28	0.82 ± 0.27	0.83 ± 0.30	0.84 ± 0.28
AP +RL + ARO + ALO	0.84 ± 0.30	0.89 ± 0.31	0.83 ± 0.29	0.86 ± 0.31	0.85 ± 0.31	0.86 ± 0.30	0.83 ± 0.28	0.82 ± 0.27	0.82 ± 0.30	0.83 ± 0.28

**Table 4.** Lung voxel motion data. Mean (minimum, maximum) overall motion (DVF) and directional motion (SI, AP, LR) in millimeters for each case, with total voxel counts included for reference.

Case	Overall DVF (mm)	SI (mm)	AP (mm)	RL (mm)	Lung_voxels_number
1	2.22 (0.00, 12.10)	2.12 (0.00, 12.03)	0.47 (0.00, 4.14)	0.21 (0.00, 2.50)	866 043
2	3.50 (0.00, 25.08)	3.29 (0.00, 24.99)	0.71 (0.00, 7.21)	0.34 (0.00, 4.51)	850 984
3	2.93 (0.00, 14.62)	2.62 (0.00, 14.51)	0.74 (0.00, 7.64)	0.48 (0.00, 4.34)	982 524
4	3.59 (0.00, 16.38)	3.46 (0.00, 16.35)	0.61 (0.00, 4.97)	0.35 (0.00, 4.26)	705 360
5	3.90 (0.00, 19.58)	3.61 (0.00, 18.68)	1.19 (0.00, 9.13)	0.24 (0.00, 3.25)	956 398
6	5.20 (0.04, 24.32)	4.36 (0.00, 22.42)	2.15 (0.00, 16.44)	0.99 (0.00, 7.66)	1015 888
7	5.92 (0.02, 24.59)	5.59 (0.00, 24.53)	1.26 (0.00, 10.42)	0.76 (0.00, 5.92)	1330 267
8	7.72 (0.02, 27.65)	6.49 (0.00, 25.50)	3.16 (0.00, 20.37)	1.10 (0.00, 7.31)	1860 464
9	1.89 (0.03, 12.68)	1.50 (0.00, 12.67)	0.87 (0.00, 11.07)	0.23 (0.00, 4.18)	838 896
10	4.18 (0.02, 21.54)	3.62 (0.00, 20.16)	1.61 (0.00, 19.01)	0.66 (0.00, 8.08)	985 815

## Data Availability

The data that support the findings of this study are openly available at the following URL/DOI: https://med.emory.edu/departments/radiation-oncology/research-laboratories/deformable-image-registration/downloads-and-reference-data/index.html, https://learn2reg.grand-challenge.org/Learn2Reg2021/ and https://learn2reg.grand-challenge.org/Learn2Reg2022/ (Richard n.d.)
